# Response of anthocyanin biosynthesis to light by strand-specific transcriptome and miRNA analysis in *Capsicum annuum*

**DOI:** 10.1186/s12870-021-03423-6

**Published:** 2022-02-22

**Authors:** Yan Zhou, Muhammad Ali Mumtaz, Yonghao Zhang, Zhuang Yang, Yuanyuan Hao, Huangying Shu, Jie Zhu, Wenlong Bao, Shanhan Cheng, Guopeng Zhu, Zhiwei Wang

**Affiliations:** 1 Key Laboratory for Quality Regulation of Tropical Horticultural Crops of Hainan Province/Engineering Research Center of the Ministry of Education for New Variety Breeding of Tropical Crop, School of Horticulture, Hainan University, Haikou, 570228 China; 2 Institute of Tropical Horticulture Research in Hainan Academy of Agricultural Sciences, Haikou, 571100 China

**Keywords:** Pepper, Anthocyanin, Transcriptome, lncRNA, miRNA, Photosensitivity

## Abstract

**Background:**

Anthocyanins have distinct biological functions in plant coloring, plant defense against strong light, UV irradiation, and pathogen infection. Aromatic hydroxyl groups and ortho-dihydroxyl groups in anthocyanins are able to inhibit free-radical chain reactions and hydroxyl radicals. Thus, anthocyanins play an antioxidative role by removing various types of ROS. Pepper is one of the solanaceous vegetables with the largest cultivation area in China. The purple-fruited pepper is rich in anthocyanins, which not only increases the ornamental nature of the pepper fruit but also benefits the human body. In this experiment, light-induced regulatory pathways and related specific regulators of anthocyanin biosynthesis were examined through integrative transcriptomic and metabolomic analysis.

**Results:**

Results revealed that delphinium 3-O-glucoside significantly accumulated in light exposed surface of pepper fruit after 48 h as compared to shaded surface. Furthermore, through strand-specific sequencing technology, 1341 differentially expressed genes, 172 differentially expressed lncRNAs, 8 differentially expressed circRNAs, and 28 differentially expressed miRNAs were identified significantly different among both surfaces. The flavonoid synthesis pathway was significantly enriched by KEGG analysis including *SHT* (XM_016684802.1), *AT-like* (XM_016704776.1), *CCoAOMT* (XM_016698340.1, XM_016698341.1), *CHI* (XM_016697794.1, XM_016697793.1), *CHS2* (XM_016718139.1), *CHS1B* (XM_016710598.1), *CYP98A2-like* (XM_016688489.1), *DFR* (XM_016705224.1), *F3’5’H* (XM_016693437.1), *F3H* (XM_016705025.1), *F3’M* (XM_016707872.1), *LDOX* (XM_016712446.1), *TCM* (XM_016722116.1) and *TCM-like* (XM_016722117.1). Most of these significantly enriched flavonoid synthesis pathway genes may be also regulated by lncRNA. Some differentially expressed genes encoding transcription factors were also identified including *MYB4-like* (XM_016725242.1), *MYB113-like* (XM_016689220.1), *MYB308-like* (XM_016696983.1, XM_016702244.1), and *EGL1* (XM_016711673.1). Three ‘lncRNA-miRNA-mRNA’ regulatory networks with *sly-miR5303*, *stu-miR5303g*, *stu-miR7997a*, and *stu-miR7997c* were constructed, including 28 differentially expressed mRNAs and 6 differentially expressed lncRNAs.

**Conclusion:**

Possible light regulated anthocyanin biosynthesis and transport genes were identified by transcriptome analysis, and confirmed by qRT-PCR. These results provide important data for further understanding of the anthocyanin metabolism in response to light in pepper.

**Supplementary Information:**

The online version contains supplementary material available at 10.1186/s12870-021-03423-6.

## Background

Flavonoids are natural products well known for their health benefits as they are considered essential components in various medicinal, pharmaceutical, and cosmetic applications [1]. Studies have shown that flavonoids such as anthocyanins have antioxidant properties [2], and play an important role in drought tolerance [3], resistance to UV-B damage [4, 5], and pathogens [6]. With the development of society, people have a stronger desire to pursue high-quality natural foods that are beneficial to health [7–9].

*Capsicum* L. is an important solanaceous vegetable with a large cultivation area globally. Pepper fruit has a broad spectrum of health benefits and a unique spicy taste that makes it an indispensable food ingredient. In addition to being rich in vitamin C, capsaicin, carotenoids, purple pepper also contains numerous secondary metabolites such as anthocyanins [10]. The biosynthesis mechanism of flavonoids is one of the most important metabolic pathways currently being studied [11]. Delphinidin-3-trans-coumaroylrutinoside-5-glucoside is the main anthocyanin in purple pepper. Anthocyanins are synthesized in the cytoplasm and are finally transported by flavonoid transporters to the vacuole for storage [7]. Taking a synthetic pathway of capsicum flavonoids as an example, phenylalanine is first catalyzed by phenylalanine ammonia-lyase to cinnamic acid. It then undergoes a series of enzyme activities, such as cinnamic acid 4-hydroxylase (C4H), 4-coumarin-CoA ligase (4Cl), ketone synthase (CHS), chalcone isomerase (CHI), and flavone 3-hydroxylase (F3H) that catalyzes the synthesis of dihydrokaempferol. Then it is catalyzed by dihydroflavonol 4-reductase (DFR) into leucopelargonidin to form colorless anthocyanins [12, 13]. Further, under the catalysis of anthocyanin synthase (ANS), colored anthocyanins are synthesized and transported by transferase to the vacuole for storage.

In most plants except radish, carrot, and potato, anthocyanin biosynthesis and accumulation are induced by light [1]. The synthesis of anthocyanins is regulated by a variety of transcription factors, such as R2R3MYB [14], basic helix-loop-helix (bHLH) [15], WD40 protein [16, 17], and WRKY [18]. Especially, MBW (MYB-bHLH-WDR) [19] protein complex play an important role in the flavonoid synthesis pathway. This study aimed to analyze light-induced anthocyanin synthetic regulation through transcriptome analysis (including mRNA and non-coding RNA). Our results identified anthocyanin biosynthesis and transport genes regulated by light and establish a strong basis for further exploring the molecular mechanism of anthocyanin synthesis and regulation in pepper.

## Results

### Changes in fruit color and anthocyanin content after light treatment

**Fig. 1:**
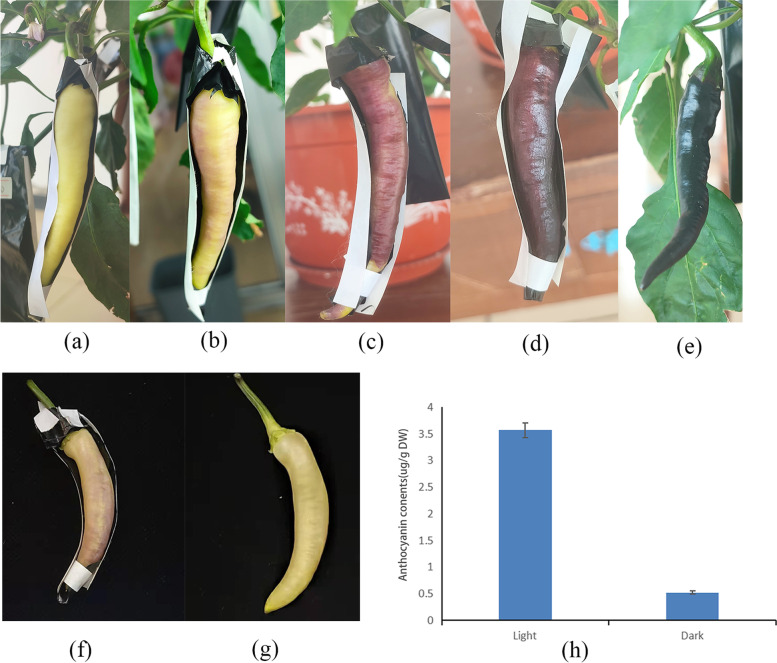
Response of the HNUCA7454 to light treatment. **a** 0 h of light, yellowish-white fruit. **b** 48 h of light, light green, and light purple fruit. **c** 72 h of light, purple fruit. **d** 7 days of light, purple-black fruit. **e** unbagged fruit. **f** 48 h of the light side. **g** 48 h of the dark side. **h** Total-Content of Anthocyanins of fruit after 48 h of light

After 48 h of different light treatment, the light-exposed surface had a light purple color (Fig. 1b), while the color of the fruit on the shaded side was still milky white without purple coloration. The total content of anthocyanin were 3.57 μg/g (freeze-dried) in the light treated and 0.523 μg /g (freeze-dried) in the dark treated pepper fruits (Fig. 1h).

### Determination of types and contents of anthocyanins by LC-MS/MS

**Fig. 2:**
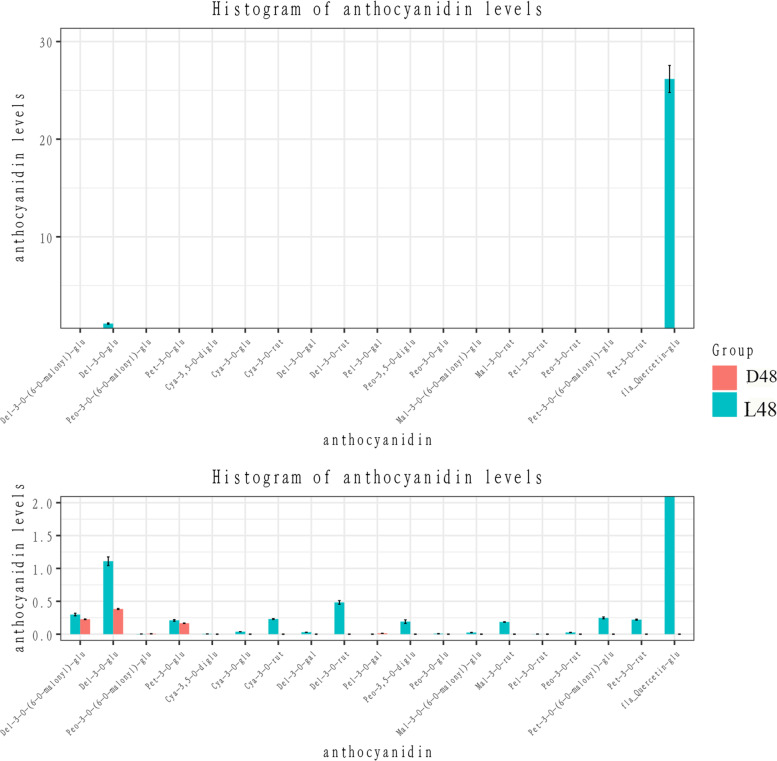
The histogram of the main anthocyanin types and contents of HNUCA7454 pepper under light and dark treatments. Note: The abscissa is the content (unit: μg/g (freeze-dried)); the ordinate is the type; the Error Bars in the figure represent standard error

LC-MS/MS was used to determine and calculate the total anthocyanin content. The total contents of L48 and D48 were 3.3104 μg/g and 0.5686 μg/g, respectively (Fig. 2), which is consistent with the results of our determination of total anthocyanin content using the pH differential method. Delphinidin 3-O-glucoside was determined as the main anthocyanins in pepper fruit. The content of Delphinidin 3-O-glucoside in L48 were 1.11 μg /g and 0.383 μg/g in D48, accounting for 33.53 and 67.41% of the total content, respectively. Delphinidin 3-O-(6′-O-malonyl)-beta-D-glucoside, Peonidin3-O-(6-O-malonyl-beta-D-glucoside), and Petunidin 3-O-glucoside were present in L48 and D48 in minor quantity and remained unchanged in different light treatments. Pelargonidin 3-O-galactoside was only present in the D48 (0.0132 μg/g). And Delphinidin 3-O-rutinoside, Cyanidin 3-O-rutinoside, Petunidin 3-O-(6-O-malonyl-beta-D-glucoside), Petunidin 3-O-rutinoside and other 13 anthocyanins were only synthesized in L48. Interestingly, a higher amount of Quercetin 3-O-glucoside was observed in L48 (26.167 μg/g). These results show that light intensity largely affects the synthesis of flavonols.

### Mapping and quality analysis of total transcriptome RNA

To explore the anthocyanins synthesis pathway in purple pepper fruits, six chain-specific RNA libraries (including mRNA, lncRNA, and circRNA) and six small RNA libraries were constructed for the transcriptome analysis of D48 and L48. For strand-specific RNA sequencing, the original readings of these six samples ranged from 10,317.48 to 1354.71356 million, resulting in RNA-Seq clean bases ranging from 15.04 to 19.88G. The Q20 percentage exceeded 97.88%, the Q30 percentage exceeded 93.71%, and the GC percentage was between 41.74 and 42.11% (Table S1). For miRNA sequencing, the raw reads of these six samples ranged from 1346 to 20.881 million, resulting in RNA-Seq clean bases of 0.673-1.044G. The percentage of Q20 exceeded 98.66%, the percentage of Q30 exceeded 95.81%, and the percentage of GC was between 47.58 and 50.46% (Table S2). For strand-specific RNA sequencing, more than 89.4% of the clean reads were successfully mapped to the reference genome, of which at least 73.36 and 13.72% of the clean reads were located uniquely and multiple times, respectively (Table S3). For miRNA sequencing, more than 90.92% of the reads were successfully mapped to the reference genome, of which at least 67.18 and 22.08% of the reads were mapped to the strand with the same reference sequence direction and the strand with the opposite direction of the reference sequence respectively (Table S4).

### Analysis of DE mRNAs

**Fig. 3:**
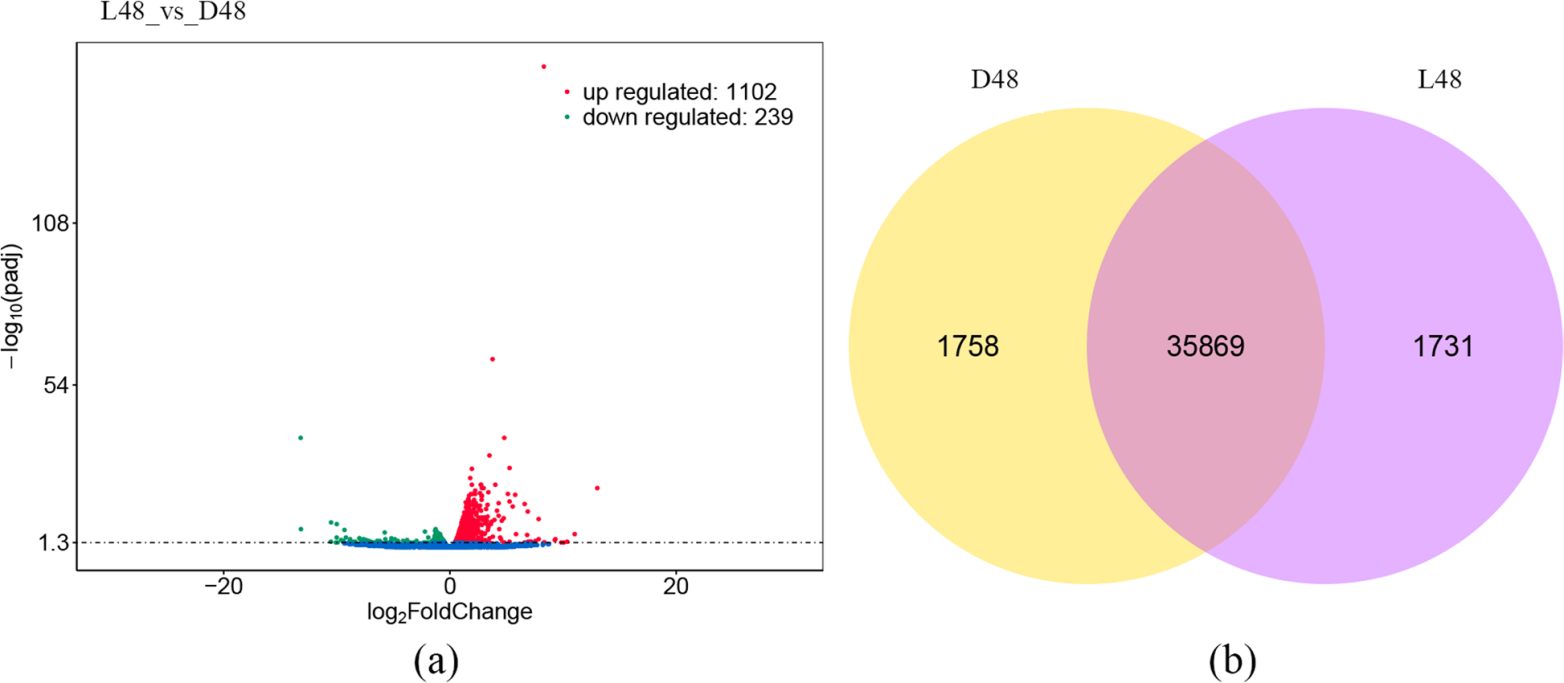
D48 and L48 mRNA analysis. **a** D48 and L48 DE mRNA volcano map. Significantly up-regulated and significantly down-regulated mRNAs are represented by red and green dots, respectively. Blue dots represent mRNAs that are not significantly different. **b** Venn diagram of the mRNA expression quantity of all D48 and L48 read count values greater than or equal to 1

From the differentially expressed (DE) mRNAs analysis between D48 and L48, we detected a total of 1341 DE mRNAs (Table S5), of which 1102 were up-regulated and 239 were down-regulated (Fig. 3a). The Venn diagram shows the number of all mRNAs expressed commonly and independently between the two groups. Most genes were expressed simultaneously in D48 and L48 (Fig. 3b).

### Analysis of GO and KEGG corresponding genes of mRNA with a significant difference under light conditions

From the GO map analysis of the differentially expressed genes (Fig. S1), biological processes that were significantly enriched include single-organism metabolic process (GO:0044710), oxidation-reduction process (GO:0055114), metabolic process (GO:0008152), small molecule metabolic process (GO:0044281), biological process (GO:0008150), nitrogen compound metabolic process (GO:1901564), nonacid metabolic process (GO:0043436), organic acid metabolic process (GO:0006082). reductionist activity (GO:0016491), Co-enzyme binding (GO:0050662), co-factor binding (GO:0048037), and catalytic activity (GO:0003824) are significantly enriched in molecular functions. Significantly enriched in cellular components are Photostatted (GO:0009521), photosynthetic membrane (GO:0034357), thyroidal (GO:0009579), thyroidal part (GO:0044436), Photostatted II (GO:0009523), lactoprotein complex (GO:0030529), thylakoids, and other parts that are significantly enriched in cellular components may also be related to the need to promote the transport of flavonoids through vesicles or transporters [7, 20, 21].

Differentially expressed mRNAs are annotated to 98 pathways from the Kyoto Encyclopedia of Genes and Genome pathway (Table S6), of which 10 pathways are significantly enriched. The significant enrichment of differentially expressed genes in the Flavonoid biosynthesis (sly00941) pathway affected pepper fruit color. In this pathway, the genes encoding SHT, shikimate O-hydroxycinnamoyl transferase-like; AT-like, acyl sugar acyltransferase 3-like; CCoAOMT, probable caffeoyl-CoA O-methyltransferase At4g26220 isoform X1; CHI, chalcone--flavanone isomerase isoform X1; CHS1B, chalcone synthase 1B; CHS2, chalcone synthase 2; CYP98A2-like, cytochrome P450 98A2-like; DFR, dihydroflavonol-4-reductase; F3’5’H, flavonoid 3′%2C5’-hydroxylase 2; F3H, naringenin%2C2-oxoglutarate 3-dioxygenase; F3’M, flavonoid 3′-monooxygenase; LDOX, leucoanthocyanidin dioxygenase [22]; TCM, trans-cinnamate 4-monooxygenase; TCM-like, trans-cinnamate 4-monooxygenase-like; were significantly up-regulated. Details of differential genes in the Flavonoid biosynthesis (sly00941) pathway are presented in Table S7.

**Fig. 4:**
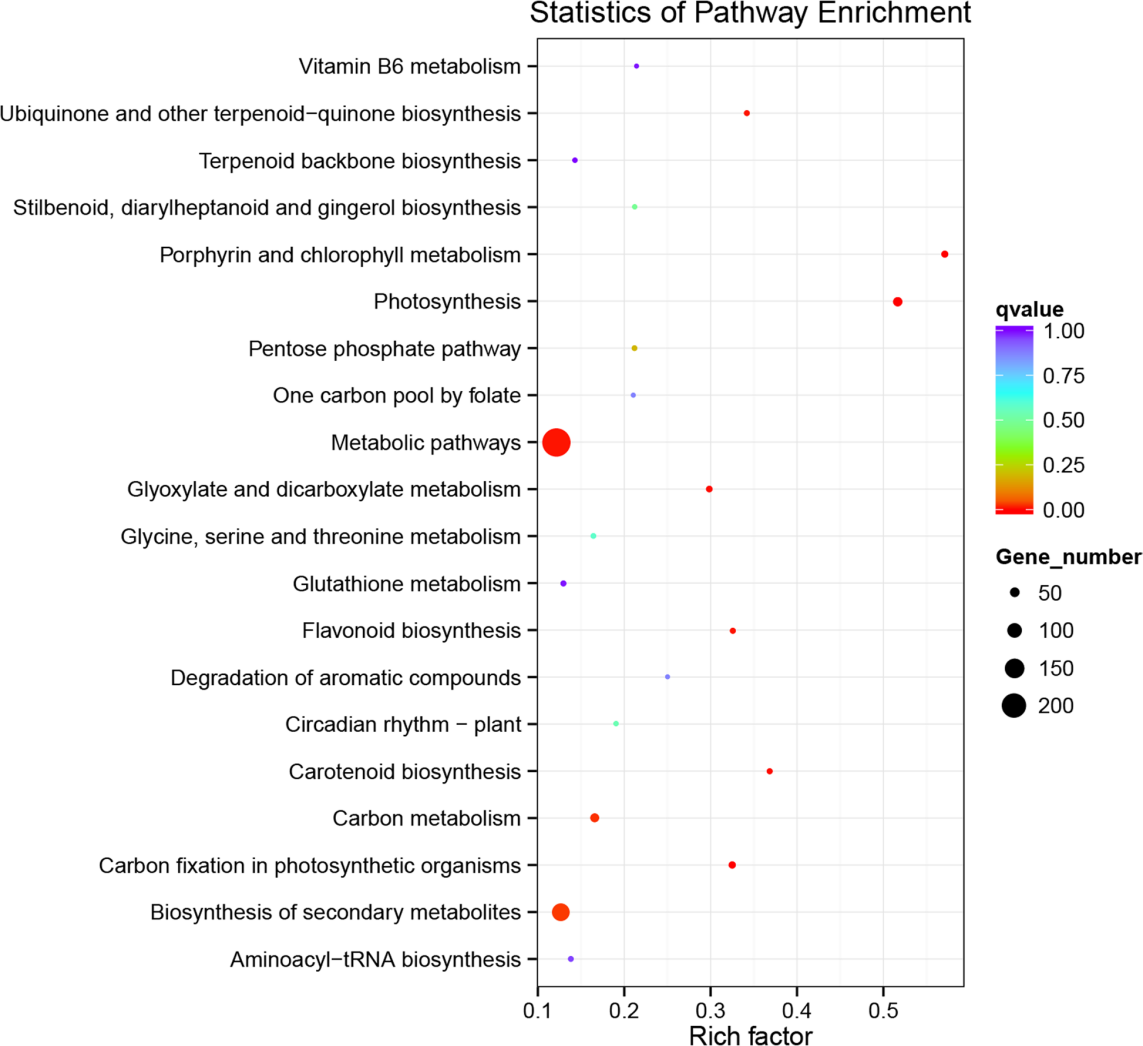
Compared with the shading surface, the differentially expressed mRNAs corresponding gene KEGG analysis in the pepper peel on the illuminated surface after 48 h of light treatment. The ordinate represents the different pathways, and the abscissa represents the number of significantly differentially expressed genes in the corresponding pathways. The colors represent different q values

**Fig. 5:**
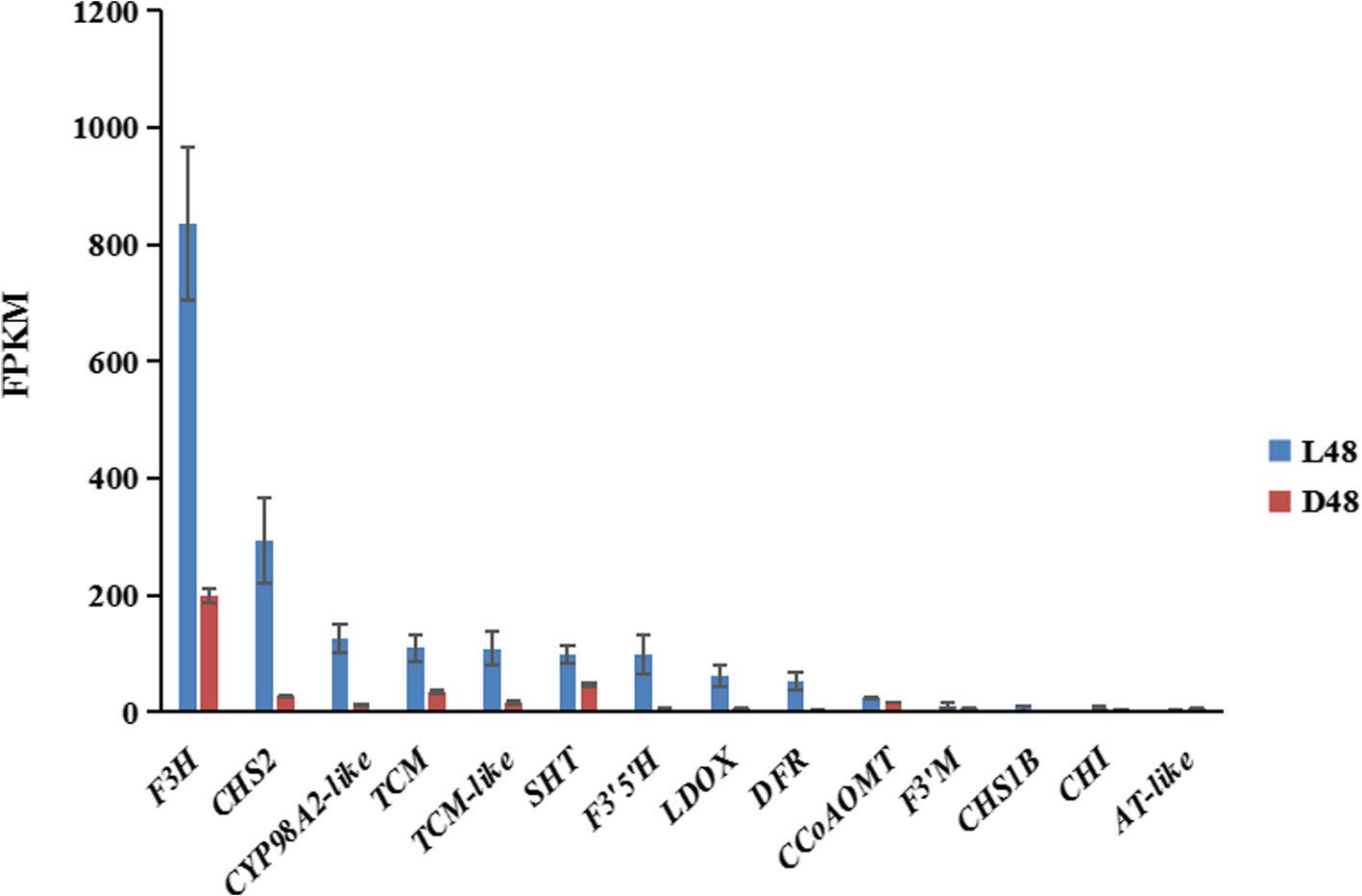
The FPKM of all genes expressed significantly differently in the sly00941 pathway. *SHT* (XM_016684802.1), *AT-like* (XM_016704776.1), *CCoAOMT* (XM_016698340.1, XM_016698341.1), *CHI* (XM_016697794.1,XM_016697793.1), *CHS2* (XM_016718139.1), *CHS1B* (XM_016710598.1), *CYP98A2-like* (XM_016688489.1), *DFR* (XM_016705224.1), *F3’5’H* (XM_016693437.1), *F3H* (XM_016705025.1), *F3’M* (XM_016707872.1), *LDOX* (XM_016712446.1), *TCM* (XM_016722116.1) and *TCM-like (XM_016722117.1)*

The early biosynthesis genes (EBGs) [23] of the flavonoid synthesis pathway include *CHS*, *CHI*, *F3H*, *F3’5’H*, and Late Biosynthesis Genes (LBGs) [23] including *DFR*, *LDOX*, were significantly up-regulated under the influence of light (Figs. 4 and 5; Fig. S2). In the pathway diagram of sly00941, we also found two cytochrome P450 enzymes, including *F3’M* (also known as *CYP706C*) and *CYP98A2*. F3’M is believed to be closely related to the flavonoid 3′, 5′ -hydroxylase (F3’5’H) [24, 25], that guides and promotes the synthesis of anthocyanins [26]. In addition, photosynthesis (sly00195; Fig. S3), glyoxylate and dicarboxylate metabolism (sly00630; Fig. S4), carbon fixation in photosynthetic organisms (sly00710; Fig. S5), porphyrin and chlorophyll metabolism (sly00860; Fig. S6), carotenoid biosynthesis (sly00906; Fig. S7), metabolic pathways (sly01100), ubiquinone and another terpenoid-quinone biosynthesis (sly00130), carbon metabolism (sly01200) and biosynthesis of secondary metabolites (sly01110) may also respond to light.

### DE mRNAs encoding transcription factors

Among the DE mRNAs encoding transcription factors obtained by transcriptome sequencing, a total of 402 DE mRNAs were predicted to be related to 53 TF families (Table S8). The top seven transcription factor family types with the highest expression abundance were *MYB* family with 30 DE mRNAs, *C2C2-GATA* family with 26 DE mRNAs, *C3H* family with 26 DE mRNAs, *mTERF* family with 25 DE mRNAs, *bZIP* family with 21 DE mRNAs, *Orphans* family with 20 DE mRNAs, *bHLH* family with 19 DE mRNAs, and the *WRKY* family has 9 DE mRNAs in response to different light conditions.

### Analysis of DE lncRNAs, DE miRNAs and DE circRNAs

Figure S8a-d shows the Volcano and Venn plots of DE lncRNAs and DE miRNAs in the light treatment group compared with the shading group, respectively. We identified 172 DE lncRNAs (88 up-regulated and 84 down-regulated) between D48 and L48. Figure S8a and b show the Volcano and Venn plots of DE lncRNAs, respectively. The detailed data of lncRNAs with significant differences between D48 and L48 are shown in Table S9.

Table S10 shows all the information of DE miRNAs. In total 28 DE miRNAs including 4 up-regulated and 24 down-regulated were detected. Details of DE circRNAs are shown in Table 1, with a total of 8 DE circRNAs (1 up-regulated and 7 down-regulated).

**Table 1:** The detailed information of DE CircRNAs between L48 and D48

circRNA ID	L48_readcount	D48_readcount	log2FoldChange	pval	padj	Regulation
novel_circ_0000040	29.00983176	0	5.2515	0.009214	0.76789	up
novel_circ_0000092	0	4.754184168	− 4.5254	0.020587	0.76789	down
novel_circ_0000320	0	7.160391679	−4.931	0.010372	0.76789	down
novel_circ_0000357	0	3.760356755	−4.1972	0.035439	0.76789	down
novel_circ_0000786	0	4.053774032	−4.1257	0.040931	0.76789	down
novel_circ_0000960	0	5.761819822	−4.7911	0.012675	0.76789	down
novel_circ_0001250	0	5.821660645	−4.6149	0.018678	0.76789	down
novel_circ_0001268	0	3.678145014	−4.1616	0.037399	0.76789	down

We use the co-localization and co-expression of DE IncRNAs and protein-coding genes to predict its biological function. Figure S9c represents the Venn diagram of the cross-analysis of DE lncRNAs, mRNAs and DEmRNAs. There are 259 up-regulated DEmRNAs with up-regulated lncRNAs, 179 down-regulated mRNAs with down-regulated lncRNAs, 43 up-regulated mRNAs with down-regulated lncRNAs, and 5 down-regulated mRNAs with up-regulated lncRNAs (Table S11). Figure S8c shows the Volcano map of DE miRNAs. There are 3 up-regulated DEmRNAs with up-regulated miRNAs, 29 down-regulated mRNAs with down-regulated miRNAs, 130 up-regulated mRNA with down-regulated miRNAs (Table S12).

### Function prediction of DE lncRNAs and DE miRNAs (Fig. S10)

The GO enrichment analysis of DE lncRNA candidate target genes showed that the mRNA of differentially expressed lncRNAs were mainly enriched in BPs. RNA metabolic process (GO:0016070), gene expression (GO:0010467), cellular nitrogen compound biosynthetic process (GO:0044271), regulation of cellular process (GO:0050794), regulation of biological process (GO:0050789), etc. were enriched.

Through the GO enrichment analysis of DE miRNA candidate target genes, the most significantly enriched MFs are ADP binding (GO:0043531), adenyl nucleotide-binding (GO:0030554), and adenyl ribonucleotide binding (GO:0032559). The most significantly enriched BPs include regulation of cellular process (GO:0050794), biological regulation (GO:0065007), regulation of biological process (GO:0050789), pigment catabolic process (GO:0046149), and chlorophyll catabolic process (GO:0015996).

We use KEGG enrichment analysis to determine the most important biochemical metabolic pathways and signal transduction pathways related to specific genes. Most mRNAs were significantly enriched in pathways including photosynthesis-antenna proteins (sly00196), porphyrin and chlorophyll metabolism (sly00860), ubiquinone and other terpenoid-quinone biosynthesis (sly00130), spliceosome (sly03040), circuit rhythm-plant (sly04712), photosynthesis (sly00195), flavonoid biosynthesis (sly00941), non-homologous end-joining (sly03450). plant-pathogen interaction (sly04626) and spliceosome (sly03040) pathways were both significant enriched.

Table S13 shows the detailed information of lncRNA and its candidate targeted mRNA in the flavonoid biosynthesis (sly00941) pathway. This pathway includes 25 genes including *SHT*, *AT-like*, *CCoAOMT*, *CHI*, *CHS1B*, *CHS2*, *CYP98A2-like*, *DFR*, *F3’5’H*, *F3H*, *F3’M*, *FLS* [27], *LDOX, TCM, TCM-like*, etc. may be regulated and expressed by lncRNA. These genes may be the key nodes for lncRNA to regulate anthocyanin synthesis. The plant-pathogen interaction (sly04626) pathway, which is significantly enriched in DE miRNAs, may be related to the stress response of plants in response to strong light. The process of plant defense against pathogens and UV damage may have a common regulatory pathway [28]. A series of biological and other non-biological reactions accompanying the synthesis of anthocyanins may make plants more resistant to the damage caused by multiple threats.

### Association network analysis

**Fig. 6:**
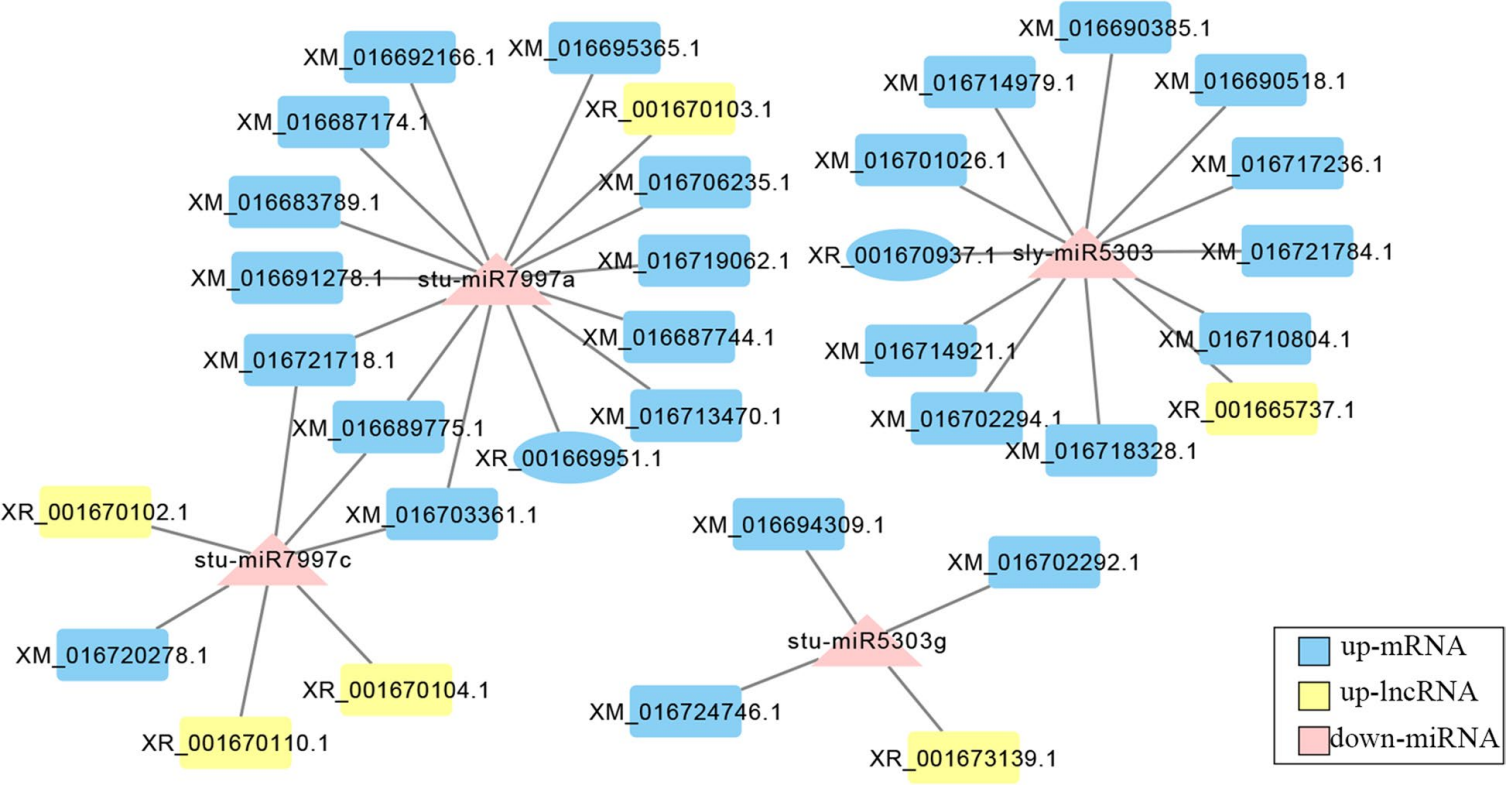
lncRNA-miRNA-mRNA correlation analysis result network

Long non-coding RNA acts as a competitive endogenous RNA to regulate gene expression. To predict interaction between miRNA and mRNA, lncRNA and miRNA. We constructed three ncRNA-miRNA-mRNA gene interaction networks which may be related to light response. Involving 28 differentially expressed mRNAs, 6 differentially expressed lncRNAs, and 4 differentially expressed miRNAs. Three regulatory networks with *sly-miR5303*, *stu-miR5303g*, *stu-miR7997a*, and *stu-miR7997c* as the core were constructed (Fig. 6).

### qRT-PCR analysis of genes related to anthocyanin metabolism in pepper

For qRT-PCR verification a total of 12 genes associated with synthetic and regulatory pathway of anthocyanin metabolism were selected. The findings revealed that all 12 DEGs expression data of qRT-PCR complied with the RNA-seq expression data (Fig. S11).

## Discussion

The enrichment of anthocyanins on the surface of plants can reduce the damage caused by strong light to plant cells and protect plant growth [4]. The isotomycin rich in purple pepper has a certain effect on reducing inflammation, promoting antioxidant enzyme activity, and preventing obesity [29]. Anthocyanins are beneficial to the health of animals and humans [30] and are considered green and healthy plant nutrients [31]. Anthocyanins are also natural food colorants and are safer for the human body.

Controlling the synthesis of anthocyanins has always been an issue of great concern. This study analyzed the anthocyanin formation under different light treatments to elaborate the molecular mechanism of light-dependent anthocyanin formation in pepper fruits. Two weeks after bagging, HNUCA7454 capsicum fruits colored quickly and anthocyanins synthesized within 48 h after receiving light. We determined the types and contents of synthetic anthocyanins on the light side and the dark side of fruit. At the same time, anthocyanin determination and transcriptome analysis were performed on the fruits under the different light treatment conditions. Eighteen types of flavonoids are synthesized on the light exposed surface. In the light exposed surface isoquercetin was identified as major accumulated anthocyanin. Whereas pelargonidin 3-O-galactoside synthesized only in the shaded surface. Furthermore, 1341 DE mRNAs, some lncRNAs and miRNAs were also identified significantly altered under different light conditions.

### The role of lncRNA and miRNA in anthocyanin synthesis and transport and other metabolic pathways

At present, the basic pathways of anthocyanin biosynthesis are relatively comprehensive, but there are few studies on anthocyanin synthesis and transportation and the mutual influence on other metabolic pathways. Noncoding RNAs (ncRNAs), can also play a vital role in regulating gene expression [32]. Recent studies have shown that miRNAs are involved in anthocyanin biosynthesis. In *A. thaliana*, increased miR156 activity promotes the accumulation of anthocyanins, whereas reduced miR156 activity directs synthesis toward flavonols [33]. The miR156 target, SQUAMOSA PROMOTER-BINDING PROTEIN-LIKE 9 (SPL9), has also been shown to suppress anthocyanin accumulation by preventing the expression of anthocyanin biosynthetic genes through destabilization of a MYB − bHLH−WD40 transcriptional activation complex [33]. In our study, we identified 6 differentially expressed lncRNAs, and 4 differentially expressed miRNAs. Among the 28 mRNAs in the ‘lncRNA-miRNA-mRNA’ interaction network, some genes are related to pathways such as Protein export, Endocytosis, Ubiquitin mediated proteolysis, Plant-pathogen interaction and Plant hormone signal transduction. Studies have shown that the accumulation of anthocyanins is related to the metabolism of plant hormones such as jasmonic acid [34, 35], abscisic acid [36], and ethylene [37], strong light [38, 39], mechanical damage, and pathogens [40]. However, the role of lncRNA in light regulation of anthocyanin biosynthesis and transport still need further verification.

### TFs response to light

Transcription factors are the key to the biosynthesis of flavonoids. The MBWs [14, 16, 19, 31] ternary complex composed of *MYB*, *bHLH*, and *WDR* has the greatest impact on the synthesis of flavonoids. It has been reported that a variety of MYBs can significantly regulate the synthesis of flavonoids [41–43]. R2R3-MYB protein promotes the accumulation of anthocyanin products in the flavonoid synthesis process of various crops such as pepper, radish [9], and tomato [44], while the effect of R3-MYB is just the opposite. The transcription factors *AtMYB11*, *AtMYB111*, and *AtMYB12* derived from *Arabidopsis* can promote the accumulation of flavonoids in tobacco [45] and delay the metabolism of narcissus flavonoids by inhibiting the biosynthesis of flavonols [29]. We identified 30 significantly different *MYB* transcription factors, including *MYB4-like* (XM_016725242.1), *MYB113-like* (XM_016689220.1), and *MYB308-like* (XM_016696983.1, XM_016702244.1). Which are the most abundant among all transcription factors. Existing studies have shown that *MYB4* [46] participates in the balanced regulation of the accumulation of anthocyanins and proanthocyanidins in mulberry fruits [47]. Another study has shown that *MYB4* plays a bidirectional role in regulating the synthesis of *Arabidopsis* flavonoids [48]. In our experimental results, *MYB4* was significantly down-regulated, showing its negative regulatory function. *MYB113* plays a positive role in the process of UV-B-induced *Arabidopsis* anthocyanin synthesis [49] and regulates anthocyanin synthesis [50].

*MYB308* is related to the biosynthesis of flavonoids [51, 52]. We found that *MYB308* has both significantly up-regulated and down-regulated genes in the process of flavonoid synthesis, indicating that it may have the same bidirectional function of regulating flavonoid synthesis as *MYB4*.

In addition, among the 19 *bHLH* transcription factors that we identified with significant differences, most importantly *EGL1* (XM_016711673.1) expression correlated with degree of anthocyanin synthesis which indicates its important role in the light regulated flavonoid biosynthesis pathway.

## Conclusions

Possible light-regulated flavonoid biosynthesis early-stage response genes, including structural genes, transcription factor families, lncRNA, miRNA, and circRNA were identified through strand specific transcriptome analysis. We determined that the main anthocyanin involved in the visible light response of HNUCA7454 pepper is delphinidin 3-O-glucosideIn this study, we identified delphinidin 3-O-glucoside as the major light regulated anthocyanin in pepper fruits. Furthermore, *SHT*, *AT-like*, *CCoAOMT*, *CHI*, *CHS1B*, *CHS2*, *CYP98A2-like*, *DFR*, *F3’5’H*, *F3H*, *F3’M*, and *FLS* were identified as major players in light-regulated anthocyanin biosynthesis. Moreover, the transcription factors including *MYB4-like*, *MYB113-like*, *MYB308-like*, and *EGL1* were probably involved in the regulation of flavonoid metabolism. Overall results from this study promote understanding of photo-regulated molecular control of anthocyanin biosynthesis and provide valuable knowledge for in depth understanding of anthocyanin molecular regulation.

## Materials and methods

### Plant materials

The pepper germplasm HNUCA7454 (*Capsicum annuum*) was collected from the germplasm bank of Hainan University, Hainan Province (20°3′ 38″ north latitude, 110°19′ 8″ east longitude). The HNUCA7454 entered the flowering stage about 80 days after sowing. Thirty flowers open on the same day were pollinated and marked simultaneously. Three days later, the pollinated flowers were bagged with a black plastic bag. After 14 days of bagging, half of the black plastic bags on the fruits were removed (Fig. 1). The fruits were treated with light for 48 h and the controlled fruits were collected and stored at − 80 °C for subsequent metabolite extraction, RNA isolation, and RNA-Seq analysis. The samples on the illuminated side and shading side were named L48 and D48, respectively. Three biological replicates were used to carry out further analysis.

### Determination of total anthocyanin

The total anthocyanin content was measured by a method previously described [53]. After grinding 0.2 g of the sample, it was added to 1.5 ml of a solution containing methanol: acetic acid (99:1, v/v), and then placed at 4 °C for overnight extraction. The extract was filtered, and then the absorbance of the extract was measured at 530, 620, and 650 nm using a DU 800 spectrophotometer (Beckman Coulter, Fullerton, California). Total anthocyanin = [(OD 530 -OD 650) -0.2 × OD 650 -OD 620).

### Determination of flavonoids by LC-ESI-MS/MS

The flavonoids in the collected samples were analyzed by Met Ware (Wuhan, China). Ultra performance liquid chromatography (UPLC) (ExionLC™AD, https://sciex.com.cn/) and tandem mass spectrometry (MS/MS) (QTRAP®6500 +, HTTP://sciex.com.cn/) (LC-ESI-MS/MS) was used for relative quantification of flavonoids [54]. The sample was freeze-dried in a vacuum and then ground and weighed out 50 mg of powder. The sample was extracted in a 500 μl extraction solution (50% methanol aqueous solution containing 0.1% hydrochloric acid) and then centrifuged to extract the supernatant. Finally, the supernatant was filtered and stored with a microporous membrane (pore size 0.22 μm) for LC-MS/MS analysis.

The liquid phase conditions mainly include: LC: ACQUITY BEH C18 (1.7 μm, 2.1 mm * 100 mm); mobile phase: phase A is ultrapure water (adding 0.1% formic acid), phase B is methanol (adding 0.1% formic acid); Degradation: B phase ratio is 0.00 min, 5%, 6.00 min increased to 50%, 12.00 min increased to 95%, maintained for 2 min, decreased 14 to 5%, equilibrated for 2 min; flow rate 0.35 ml/min; column temperature is 40 °C. The injection volume is 2 μl.

Mass spectrometry conditions mainly include Electrospray Ionization (ESI) temperature 550 °C, mass spectrometry voltage 5500 V in positive ion mode, and Curtain Gas (CUR) 35 psi. In Q-Trap 6500+, each ion pair is scanned based on the optimized declustering voltage (Declustering Potential, DP) and collision energy (Collision Energy, CE).

### RNA isolation and library preparation

The samples were analyzed and processed by Novogene Bioinformatics Technology Co. Ltd. (Beijing, China). The total RNA in the sample was extracted with Trizol Reagent (Invitrogen, Carlsbad, CA, USA), and then evaluated by agarose gel electrophoresis and RNA Nano 6000 detection kit (Agilent Technologies, CA, USA) [55]. The Illumina Hiseq platform (Illumina, USA) was used to generate a sample library, and the samples had three biological replicates. Sequencing libraries were generated using NEBNext®Multiplex Small RNA Library Prep Set for Illumina® (NEB, USA.). NEB 3′ SR Adaptor was directly and specifically ligated to 3′ end of miRNA, siRNA, and piRNA. Connect the 5 ‘end adapter to the 5’ end of miRNA, siRNA and piRNA. The first cDNA strand was then synthesized using M-MuLV Reverse Transcriptase (RNase H^−^). After PCR amplification DNA fragments corresponding to 140 ~ 160 bp were recovered, library quality was assessed on the Agilent Bioanalyzer 2100 system [56]. The reference genome was downloaded from (https://www.ncbi.nlm.nih.gov/genome/10896).

### Differential expression analysis

DE mRNA and DE lncRNA were measured with the aid of Ballgown. DESeq2 is used to identify DE miRNA and DE circRNA. Genes with a *P*-value < 0.05 are considered to be differentially expressed. Perform ontology (GO) annotation on the GO SEQ R software package, classify genes according to biological processes (BP), cell components (CC), and molecular functions (MF), and predict their functions. KEGG (Kyoto Encyclopedia of Genes and Genomes) is the main public database related to this pathway (https://www.genome.jp/kegg/;20-04-2018). Kobas v2.0 software provides help for the enrichment analysis of DE mRNA corresponding genes and DElncRNA targeted genes detected in the KEGG pathway. The transcription factor prediction in this experiment was realized by iTALK 1.2 software.

### Construction of competitive endogenous RNA regulatory network

According to the hypothesis of competitive endogenous RNA (ceRNA), lncRNA containing one or more miRNA response elements (MRE) in common can compete with mRNA to bind to miRNA to regulate expression. Cytoscape 3.7.1 software was used to construct the regulatory network between lncRNA-miRNA-mRNA.

### RNA extraction and quantitative reverse transcription-PCR

RNA prep Pure Plant Plus Kit (Tian Gen, Beijing, China) was used to extract total RNA from pepper peel samples. The integrity and purity of RNA were determined by agarose gel electrophoresis and NANODROP 2000 spectrophotometer (Thermo Fisher Scientific, MA, USA), and then quantified before use. The RNA samples were reverse-transcribed to synthesize cDNA by Fast King gDNA Dispelling RT SuperMix (Tian Gen, Beijing, China). Quantitative real-time PCR was performed using the Light Cycler 96 Real-Time PCR System (Roche, Switzerland) instrument. Evaluate the relative expression level of the target gene based on the 2-ΔΔCt method. Ubi-3 was selected as the internal reference gene to balance the differences between different samples. The primer information for qRT-PCR is given in Table S14.

## Supplementary Information

**Additional file 1: Table S1.** Quality evaluation of sample sequencing output data for strand-specific RNA libraries.

**Additional file 2: Table S2.** Quality evaluation of sample sequencing output data for small RNA libraries.

**Additional file 3: Table S3.** Reads of strand-specific RNA libraries and reference genome comparison list.

**Additional file 4: Table S4.** Reads of small RNA libraries and reference genome comparison list.

**Additional file 5: Table S5.** The detailed information of DE mRNAs.

**Additional file 6: Table S6.** KEGG analysis results of DE mRNA corresponding genes.

**Additional file 7: Table S7.** Detailed information of differential genes in the Flavonoid biosynthesis pathway.

**Additional file 8: Table S8.** DE mRNAs encoding transcription factor.

**Additional file 9: Table S9.** The detailed information of DE lncRNAs betweenL48 and D48.

**Additional file 10: Table S10.** The detailed information of DE miRNAs between L48 and D48.

**Additional file 11: Table S11.** The detailed information between DE lncRNA and its targeted mRNA.

**Additional file 12: Table S12.** The information between DE miRNA and its DE targeted mRNA (up-regulation).

**Additional file 13: Table S13.** The detailed information of lncRNA and its targeted mRNA in the Flavonoid biosynthesis (sly00941) pathway.

**Additional file 14: Table S14.** The primers information for qRT-PCR of genes related to anthocyanin synthesis.

**Additional file 15: Figure S1.** Compared with the shading surface, the DE mRNAs in the pepper peel on the shining surface after 48 h of light treatment corresponds to the GO classification of the gene. The abscissa is the name of the functional classification of gene enrichment. Followed by biological processes (BPs), cellular components (CCs), and molecular functions (MFs). The ordinate is the number and proportion of genes enriched in this function.

**Additional file 16: Figure S2.** Compared with the light-shielded surface, the KEGG analysis of the differentially expressed mRNAs corresponding genes in the pepper peel on the light-treated surface enriched the “Flavonoid biosynthesis” pathway. KO nodes in the pathway whose expression levels are up-regulated are marked with a red box, and those with up-regulated and down-regulated genes are marked with a yellow box. AT, acyl sugar acyltransferase 3-like; CCoAOMT, probable caffeoyl-CoA O-methyltransferase At4g26220 isoform X1; CHI, chalcone--flavanone isomerase isoform X1; CHS1B, chalcone synthase 1B; CYP98A2-like, cytochrome P450 98A2-like; DFR, dihydroflavonol-4-reductase; F3’5’H, flavonoid 3′%2C5’-hydroxylase 2; F3H, naringenin%2C2-oxoglutarate 3-dioxygenase; F3’M, flavonoid 3′-monooxygenase; LDOX, leucoanthocyanidin dioxygenase; TCM, trans-cinnamate 4-monooxygenase

**Additional file 17: Figure S3.** The photosynthesis pathway enriched by KEGG analysis of DE mRNA corresponding genes.

**Additional file 18: Figure S4.** The glyoxylate and dicarboxylate metabolism pathway enriched by KEGG analysis of DE mRNA corresponding genes.

**Additional file 19: Figure S5.** The carbon fixation in photosynthetic organisms pathway enriched by KEGG analysis of DE mRNA corresponding genes.

**Additional file 20: Figure S6.** The porphyrin and chlorophyll metabolism pathway enriched by KEGG analysis of DE mRNA corresponding genes.

**Additional file 21: Figure S7.** The carotenoid biosynthesis pathway enriched by KEGG analysis of DE mRNA corresponding genes.

**Additional file 22: Figure S8.** D48 and L48 lncRNA and miRNA analysis. **a** Volcano map of lncRNAs of D48 and L48. Significantly differentially expressed lncRNAs are represented by red dots (up-regulated) and green dots (down-regulated), while non-differentially expressed lncRNAs are represented by blue dots. **b** Venn graph of the number of lncRNAs with reading count values greater than or equal to 1 for D48 and L48. **c** Volcano map of miRNAs of D48 and L48. Significantly differentially expressed miRNAs are represented by red dots (up-regulated) and green dots (down-regulated), while non-differentially expressed genes are represented by blue dots. **d** Venn map of the number of miRNAs with reading count value greater than or equal to 1 for D48 and L48.

**Additional file 23: Figure S9.** CircRNAs, DE lncRNA targeted mRNA, DE miRNA targeted mRNA analysis of D48 and L48. **a** Volcano plot of circRNAs of D48 and L48. Significantly differentially expressed circRNAs are represented by red dots (up-regulated) and green dots (down-regulated), while non-differentially expressed are represented by blue dots. **b** Venn map of all circRNAs with reading count values greater than or equal to 1 for D48 and L48. **c** Venn diagram of the intersection analysis between DE lncRNA targeted mRNA and DE mRNA. **d** Venn diagram of the intersection analysis between DE miRNA targeted mRNA and DE mRNA.

**Additional file 24: Figure S10. a** and **b** GO enrichment of DE lncRNAs and miRNAs on the illuminated surface of HNUCA7454 pepper after 48 h of light treatment. The ordinate is the rich GO item, and the abscissa is the number of differentially expressed genes in the item and its proportion. Different colors are used to distinguish biological processes and molecular functions. **c** and **d** are the scatter plots of KEGG enrichment of lncRNAs and miRNAs on the illuminated surface of HNUCA7454 pepper. The vertical axis represents the pathname, and the horizontal axis represents the richness factor. The size of the dot indicates the number of differentially expressed genes in the pathway, and the color of the dot corresponds to the range of different q values.

**Additional file 25: Figure S11.** qRT-PCR analysis of genes related to anthocyanin metabolism of L48 and D48. **a** Relative expression analysis of synthetic genes. *SHT* (XM_016684802.1), *AT-like* (XM_016704776.1), *CHS1B* (XM_016710598.1), *CYP98A2-like* (XM_016688489.1), *DFR* (XM_016705224.1), *F3’5’H* (XM_016693437.1), *F3H* (XM_016705025.1), *F3’M* (XM_016707872.1), *LDOX* (XM_016712446.1). **b** Relative expression analysis of regulatory genes. *MYB113-like* (XM_016689220.1), *MYB308like-1* (XM_016696983.1), *MYB308like-2* (XM_016702244.1), The x-axis represents the different treatments, the y-axis represents the relative gene expression level and RNA-seq FPKM. Error bars indicate the standard error of the mean (*n* = 3).

## Data Availability

All the original read data have been uploaded to the NCBI serial read file (SRA, https://submit.ncbi.nlm.nih.gov/subs/sra/; this SRA submission will be published on or on 28-05-2025 Issued at the time). The accession numbers are SRR14710538 (L15QN1), SRR14710543 (D15QN1), SRR14710537 (L15QN2), SRR14710536 (L15QN3), SRR14710539 (D15QN3), SRR14710542 (D15QN2), SRR14710532 (L15QN1_raw), SRR14710533 (D15QN3_raw), SRR14710540 (L15QN3_raw), SRR14710535 (D15QN1_raw), SRR14710541 (L15QN2_raw), SRR14710534 (D15QN2_raw). Other results mentioned in this paper are shown in the article and supplementary figures and tables.

## Abbreviations

DEGs: Differentially expressed genes
RNA-seq: RNA sequencing technology
lncRNA: Long non-coding RNA
GO: Gene Ontology
KEGG: Kyoto Encyclopedia of Genes and Genome
ceRNA: Competing endogenous RNA
TFs: Transcription Factors
AT: Acyl sugar acyltransferase 3-like
CCoAOMT: Probable caffeoyl-CoA O-methyltransferase At4g26220 isoform X1
CHI: Chalcone--flavanone isomerase isoform X1
CHS1B: Chalcone synthase 1B
CYP98A2-like: Cytochrome P450 98A2-like
DFR: Dihydroflavonol-4-reductase
F3’5’H: Flavonoid 3′%2C5’-hydroxylase 2
F3H: Naringenin%2C2-oxoglutarate 3-dioxygenase
F3’M: Flavonoid 3′-monooxygenase
LDOX: Leucoanthocyanidin dioxygenase
TCM: Trans-cinnamate 4-monooxygenase
