# Supplementary figures and images for: Response of anthocyanin biosynthesis to light by strand-specific transcriptome and miRNA analysis in Capsicum annuum
Source: BMC Plant Biol. 2022 Feb 22;22:79. doi: 10.1186/s12870-021-03423-6 (PMC8862587; doi:10.1186/s12870-021-03423-6)

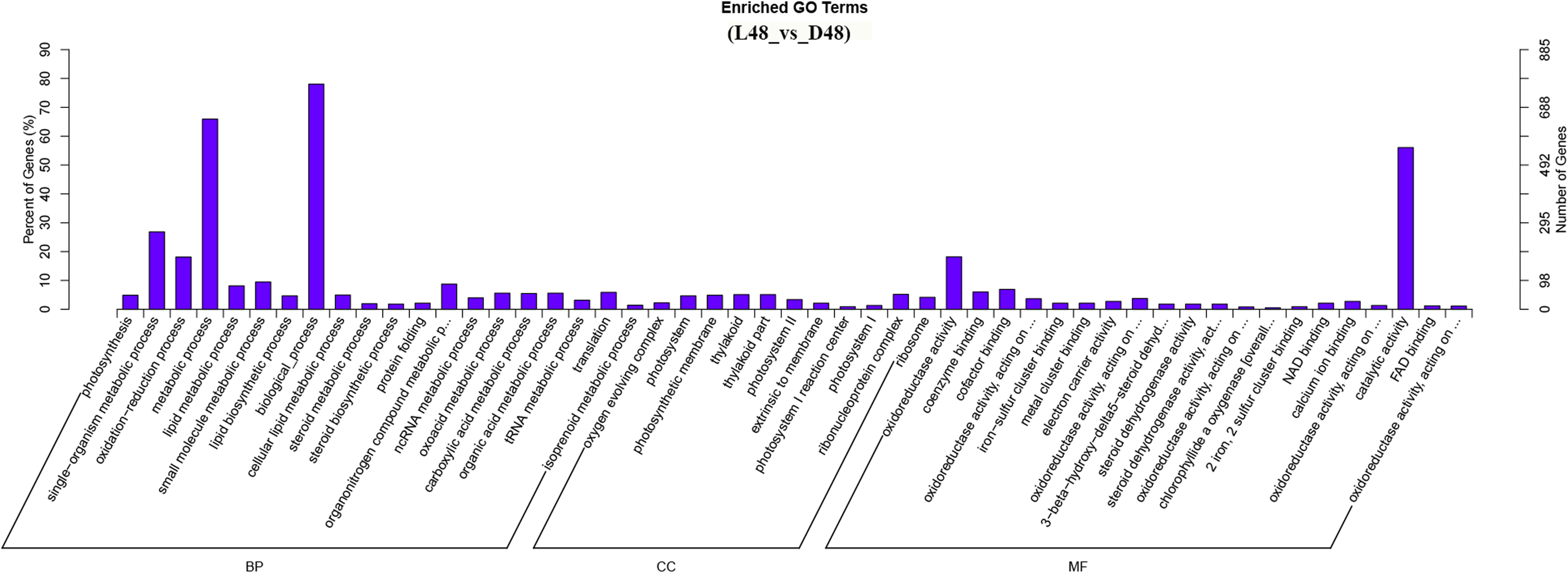

Supplement: Supplementary file 15 — Additional file 15: Figure S1. Compared with the shading surface, the DE mRNAs in the pepper peel on the shining surface after 48 h of light treatment corresponds to the GO classification of the gene. The abscissa is the name of the functional classification of gene enrichment. Followed by biological processes (BPs), cellular components (CCs), and molecular functions (MFs). The ordinate is the number and proportion of genes enriched in this function. [file 12870_2021_3423_MOESM15_ESM.tif]

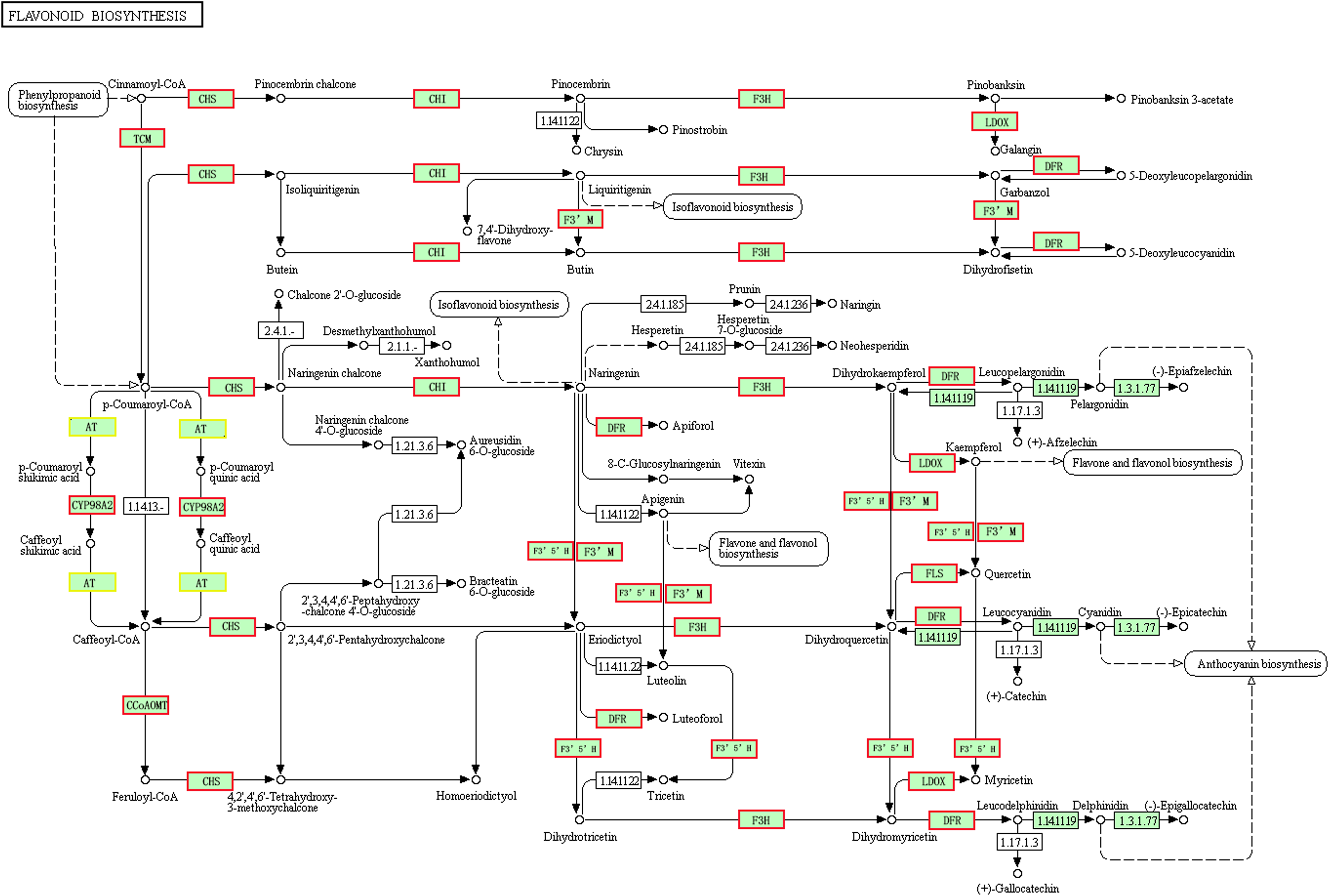

Supplement: Supplementary file 16 — Additional file 16: Figure S2. Compared with the light-shielded surface, the KEGG analysis of the differentially expressed mRNAs corresponding genes in the pepper peel on the light-treated surface enriched the “Flavonoid biosynthesis” pathway. KO nodes in the pathway whose expression levels are up-regulated are marked with a red box, and those with up-regulated and down-regulated genes are marked with a yellow box. AT, acyl sugar acyltransferase 3-like; CCoAOMT, probable caffeoyl-CoA O-methyltransferase At4g26220 isoform X1; CHI, chalcone--flavanone isomerase isoform X1; CHS1B, chalcone synthase 1B; CYP98A2-like, cytochrome P450 98A2-like; DFR, dihydroflavonol-4-reductase; F3’5’H, flavonoid 3′%2C5’-hydroxylase 2; F3H, naringenin%2C2-oxoglutarate 3-dioxygenase; F3’M, flavonoid 3′-monooxygenase; LDOX, leucoanthocyanidin dioxygenase; TCM, trans-cinnamate 4-monooxygenase [file 12870_2021_3423_MOESM16_ESM.tif]

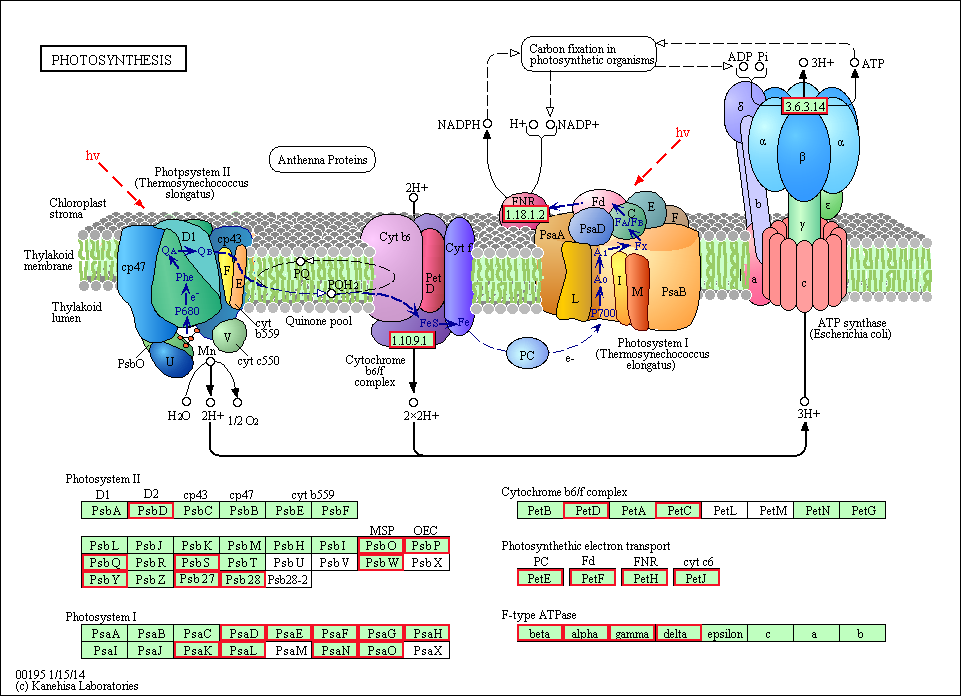

Supplement: Supplementary file 17 — Additional file 17: Figure S3. The photosynthesis pathway enriched by KEGG analysis of DE mRNA corresponding genes. [file 12870_2021_3423_MOESM17_ESM.tif]

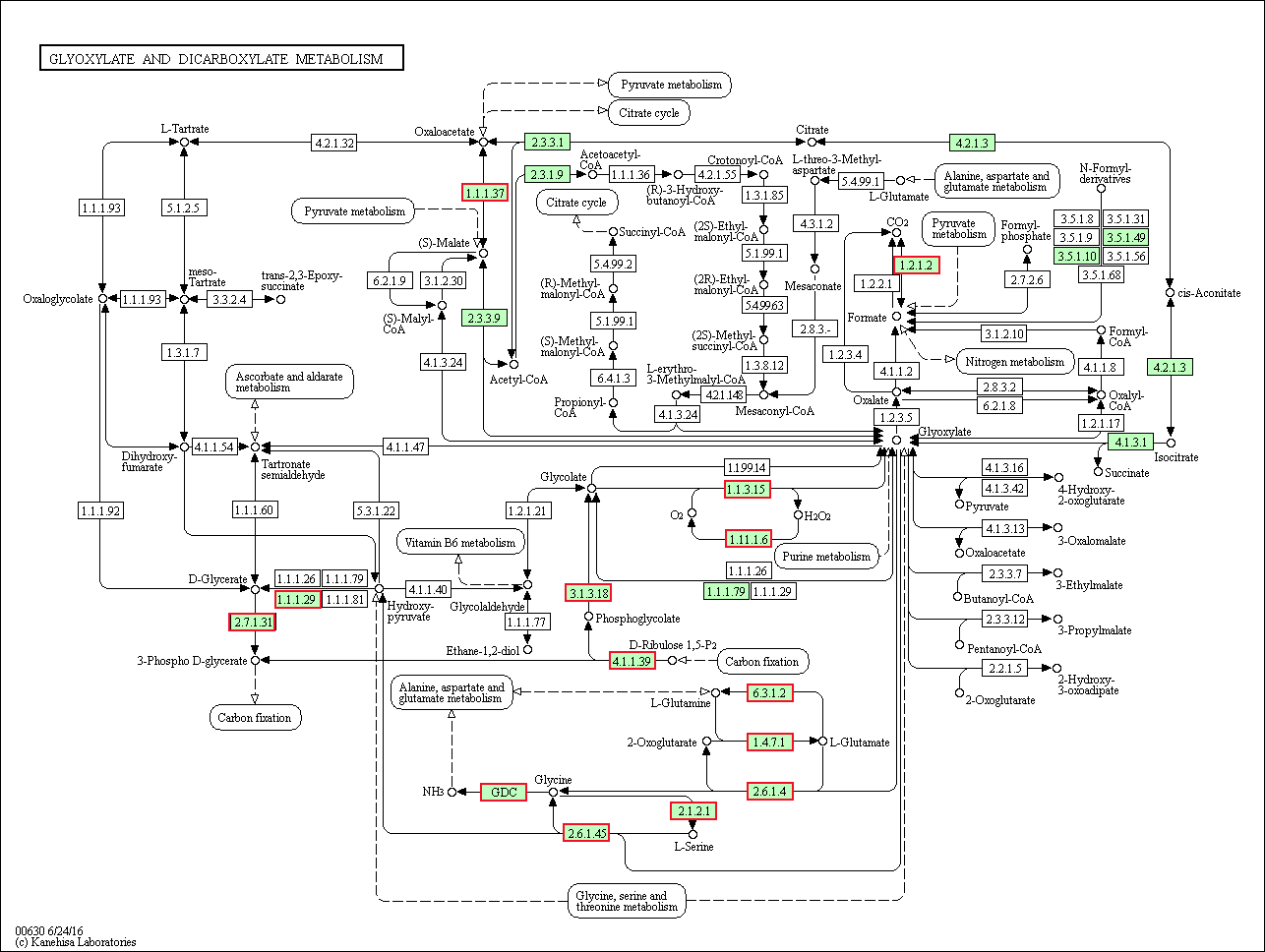

Supplement: Supplementary file 18 — Additional file 18: Figure S4. The glyoxylate and dicarboxylate metabolism pathway enriched by KEGG analysis of DE mRNA corresponding genes. [file 12870_2021_3423_MOESM18_ESM.tif]

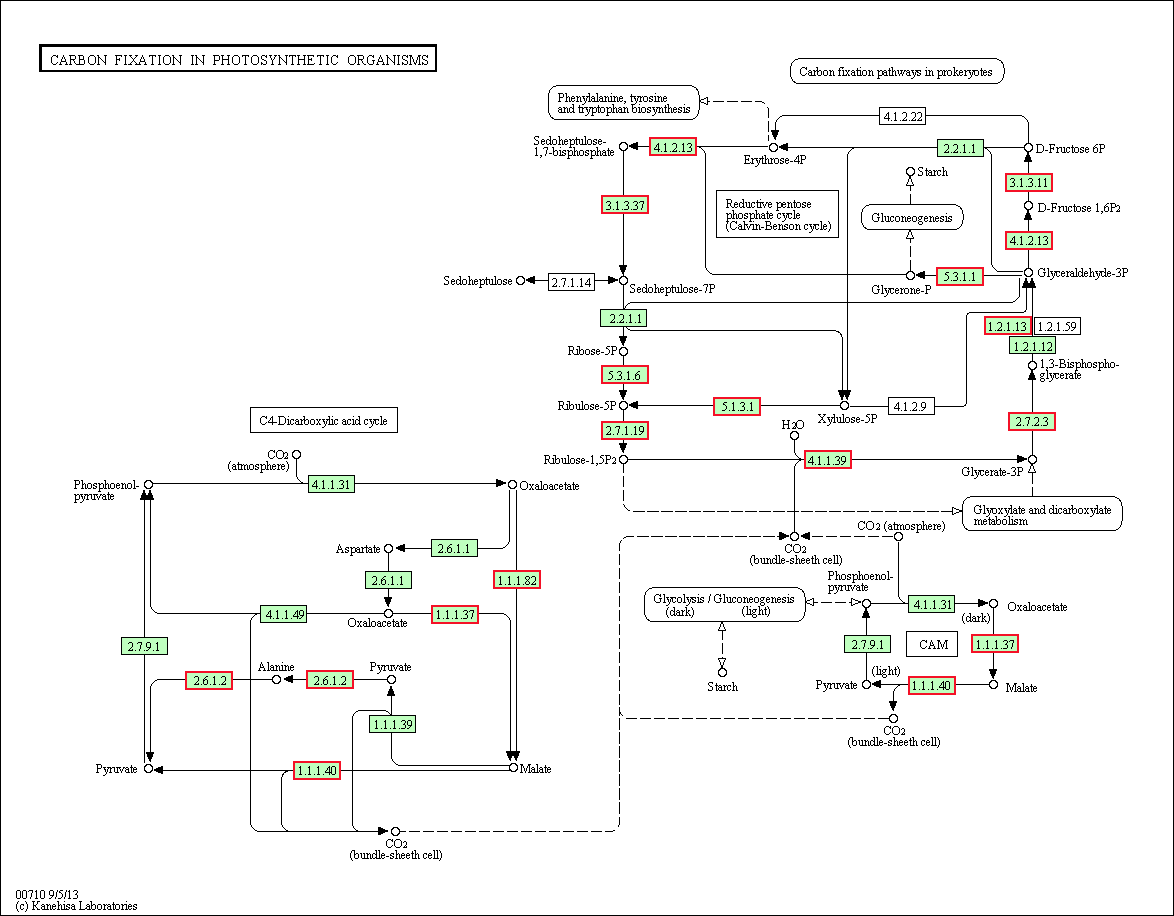

Supplement: Supplementary file 19 — Additional file 19: Figure S5. The carbon fixation in photosynthetic organisms pathway enriched by KEGG analysis of DE mRNA corresponding genes. [file 12870_2021_3423_MOESM19_ESM.tif]

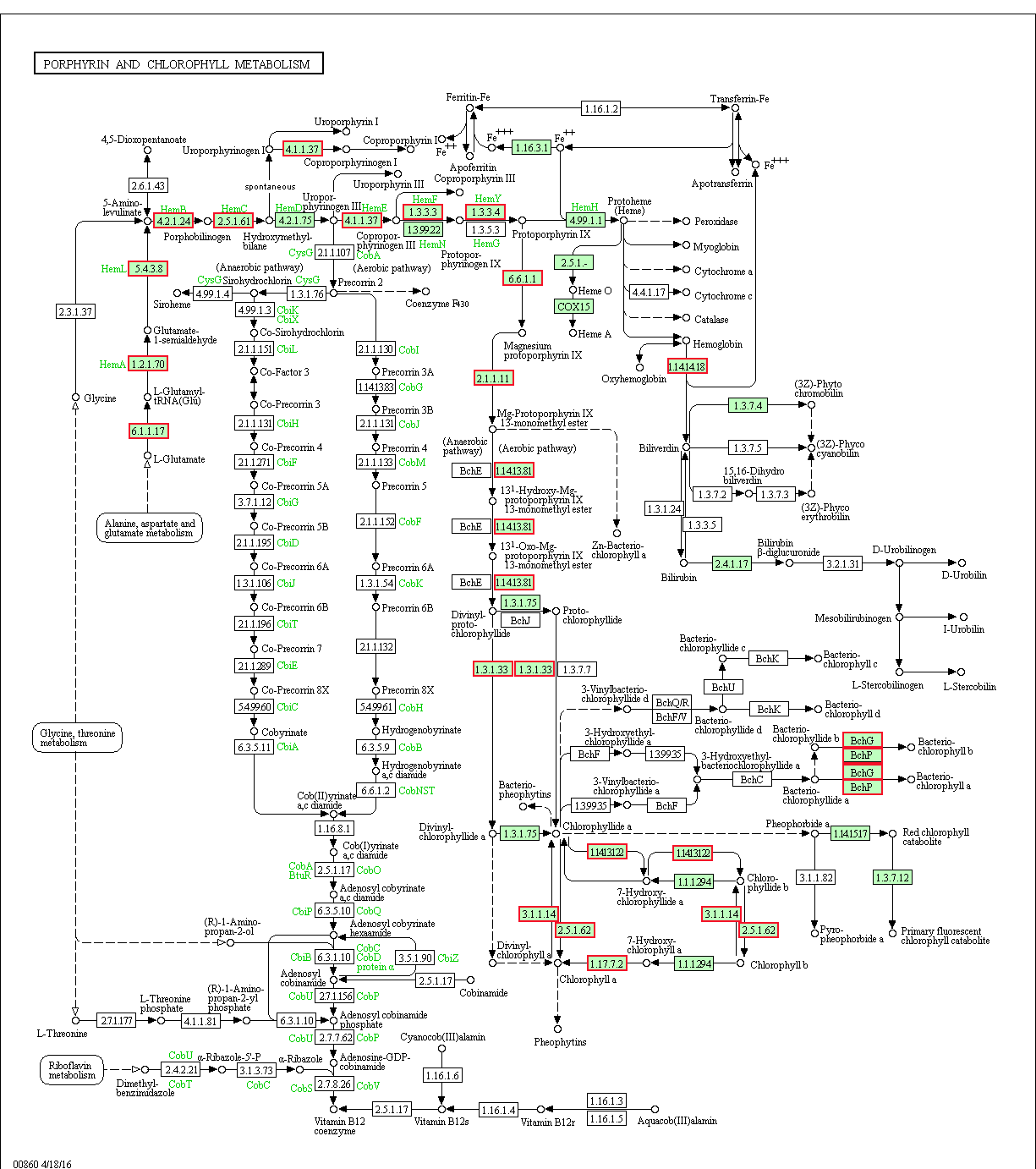

Supplement: Supplementary file 20 — Additional file 20: Figure S6. The porphyrin and chlorophyll metabolism pathway enriched by KEGG analysis of DE mRNA corresponding genes. [file 12870_2021_3423_MOESM20_ESM.tif]

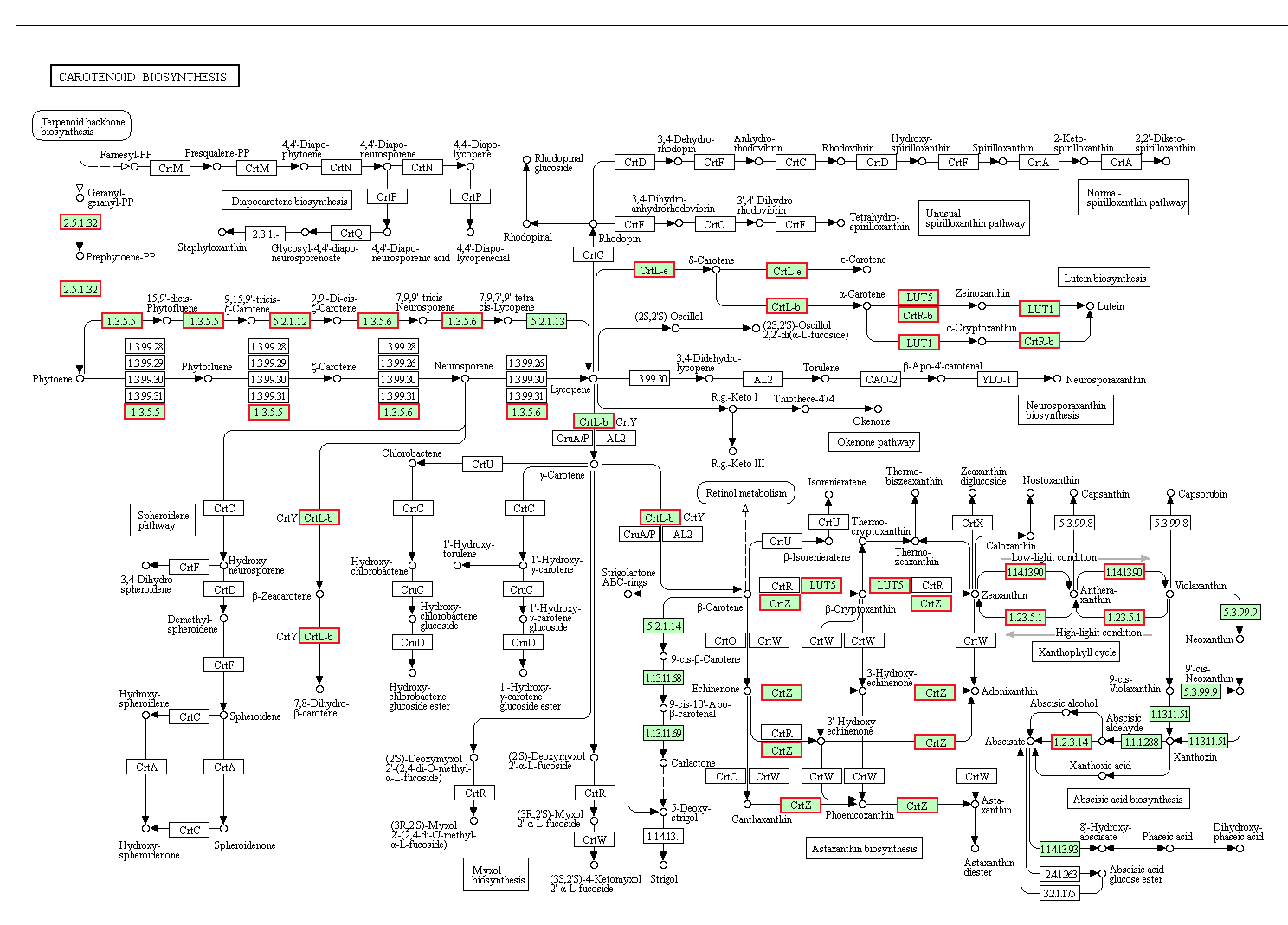

Supplement: Supplementary file 21 — Additional file 21: Figure S7. The carotenoid biosynthesis pathway enriched by KEGG analysis of DE mRNA corresponding genes. [file 12870_2021_3423_MOESM21_ESM.tif]

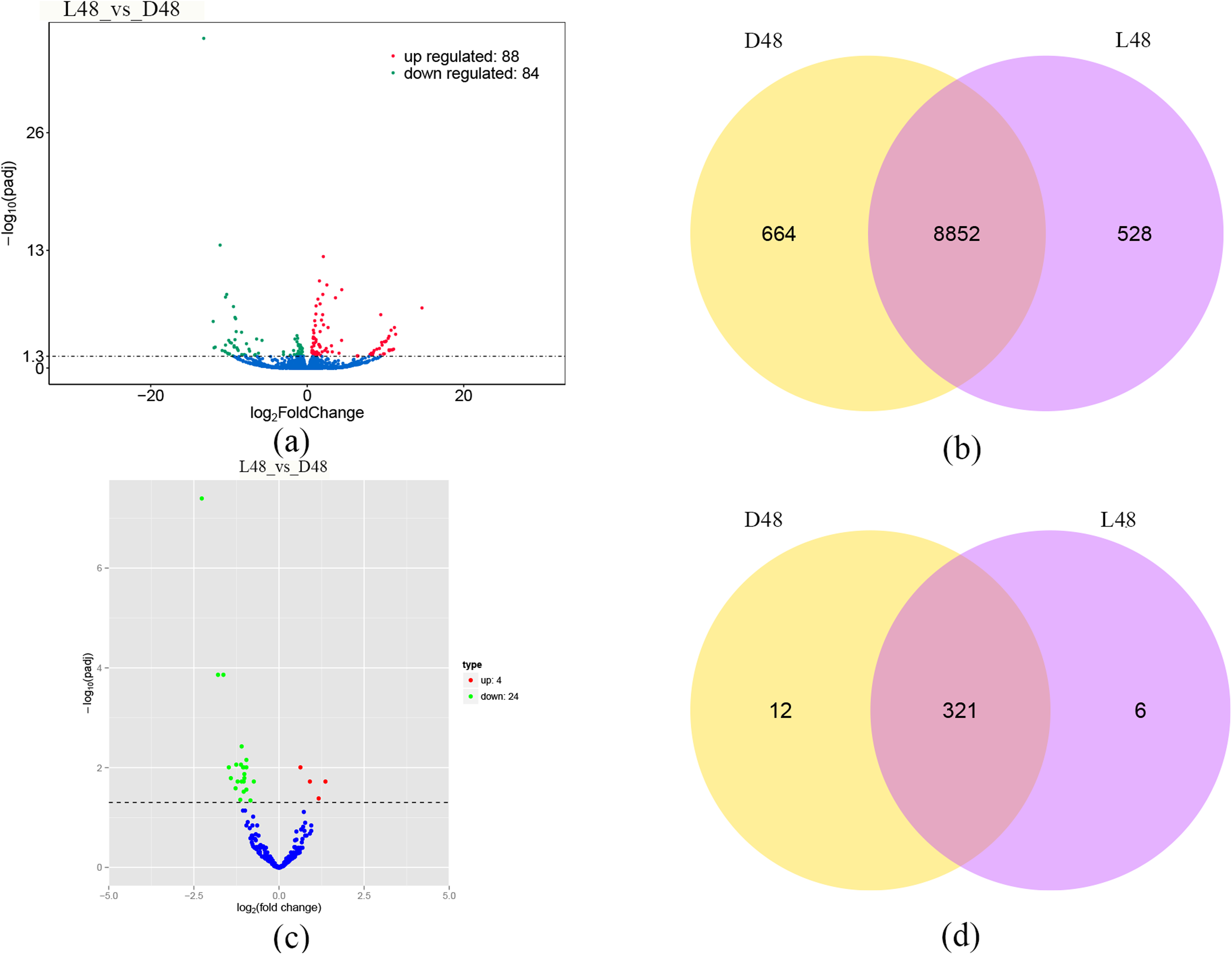

Supplement: Supplementary file 22 — Additional file 22: Figure S8. D48 and L48 lncRNA and miRNA analysis. a Volcano map of lncRNAs of D48 and L48. Significantly differentially expressed lncRNAs are represented by red dots (up-regulated) and green dots (down-regulated), while non-differentially expressed lncRNAs are represented by blue dots. b Venn graph of the number of lncRNAs with reading count values greater than or equal to 1 for D48 and L48. c Volcano map of miRNAs of D48 and L48. Significantly differentially expressed miRNAs are represented by red dots (up-regulated) and green dots (down-regulated), while non-differentially expressed genes are represented by blue dots. d Venn map of the number of miRNAs with reading count value greater than or equal to 1 for D48 and L48. [file 12870_2021_3423_MOESM22_ESM.tif]

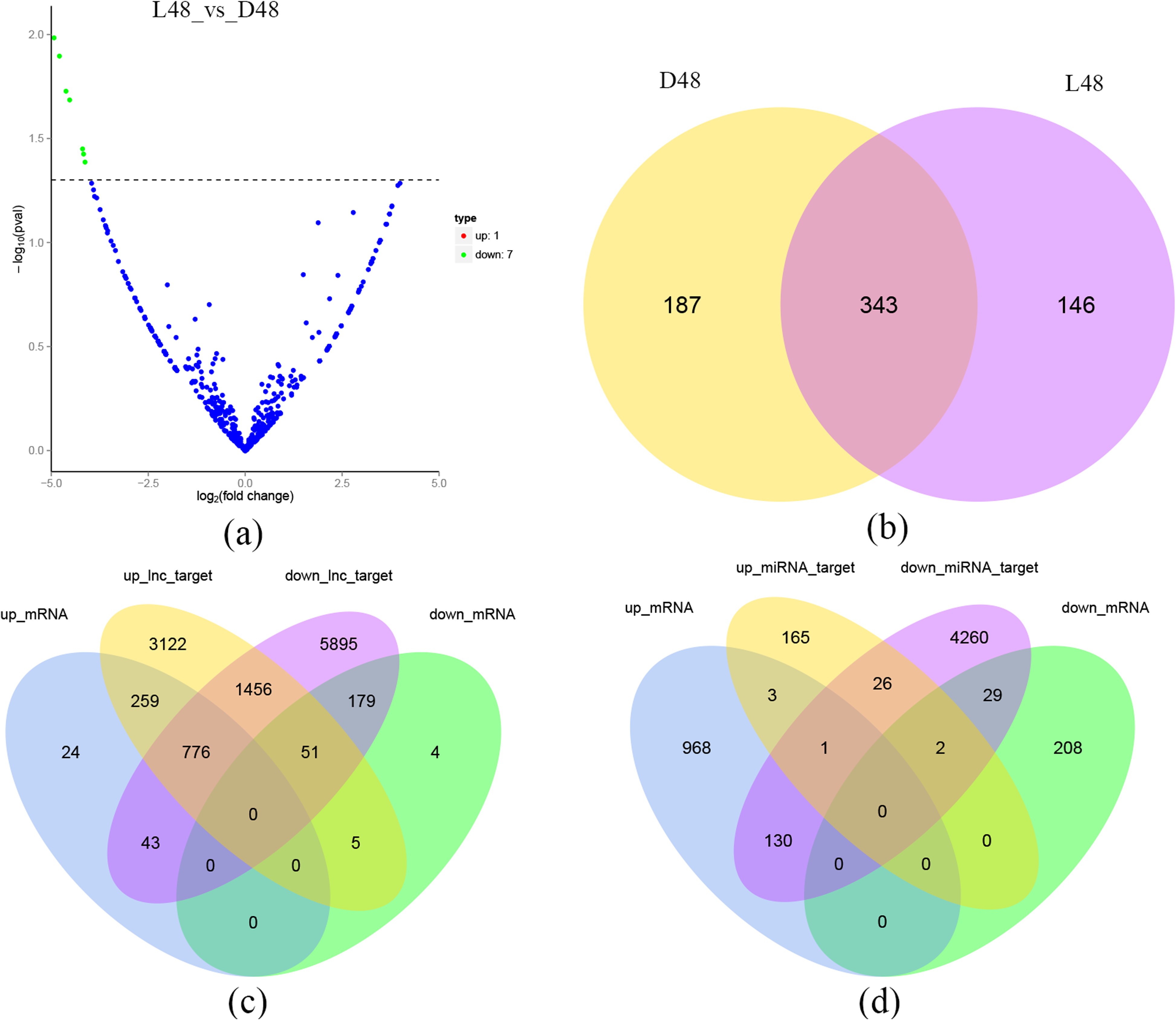

Supplement: Supplementary file 23 — Additional file 23: Figure S9. CircRNAs, DE lncRNA targeted mRNA, DE miRNA targeted mRNA analysis of D48 and L48. a Volcano plot of circRNAs of D48 and L48. Significantly differentially expressed circRNAs are represented by red dots (up-regulated) and green dots (down-regulated), while non-differentially expressed are represented by blue dots. b Venn map of all circRNAs with reading count values greater than or equal to 1 for D48 and L48. c Venn diagram of the intersection analysis between DE lncRNA targeted mRNA and DE mRNA. d Venn diagram of the intersection analysis between DE miRNA targeted mRNA and DE mRNA. [file 12870_2021_3423_MOESM23_ESM.tif]

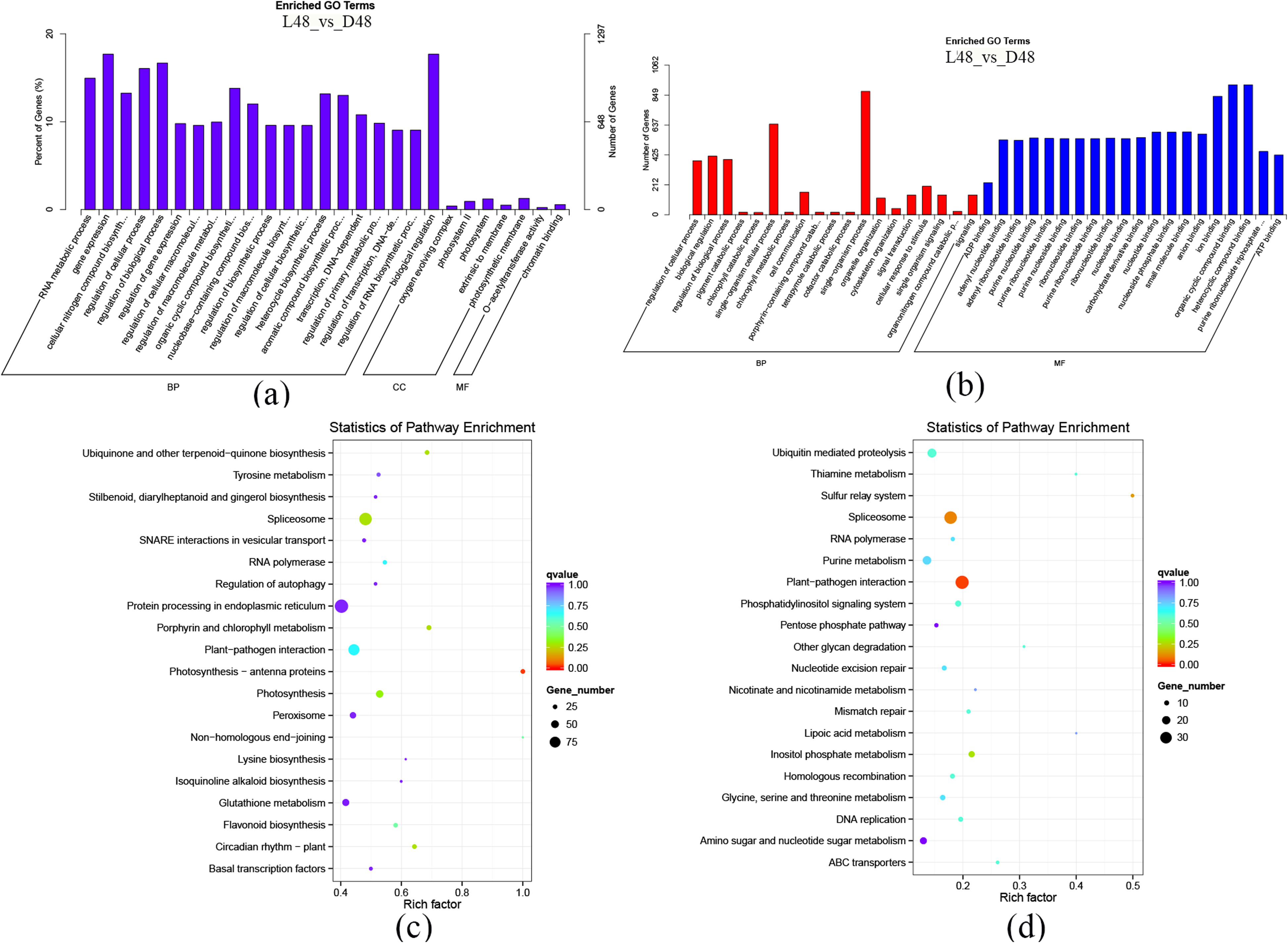

Supplement: Supplementary file 24 — Additional file 24: Figure S10. a and b GO enrichment of DE lncRNAs and miRNAs on the illuminated surface of HNUCA7454 pepper after 48 h of light treatment. The ordinate is the rich GO item, and the abscissa is the number of differentially expressed genes in the item and its proportion. Different colors are used to distinguish biological processes and molecular functions. c and d are the scatter plots of KEGG enrichment of lncRNAs and miRNAs on the illuminated surface of HNUCA7454 pepper. The vertical axis represents the pathname, and the horizontal axis represents the richness factor. The size of the dot indicates the number of differentially expressed genes in the pathway, and the color of the dot corresponds to the range of different q values. [file 12870_2021_3423_MOESM24_ESM.tif]

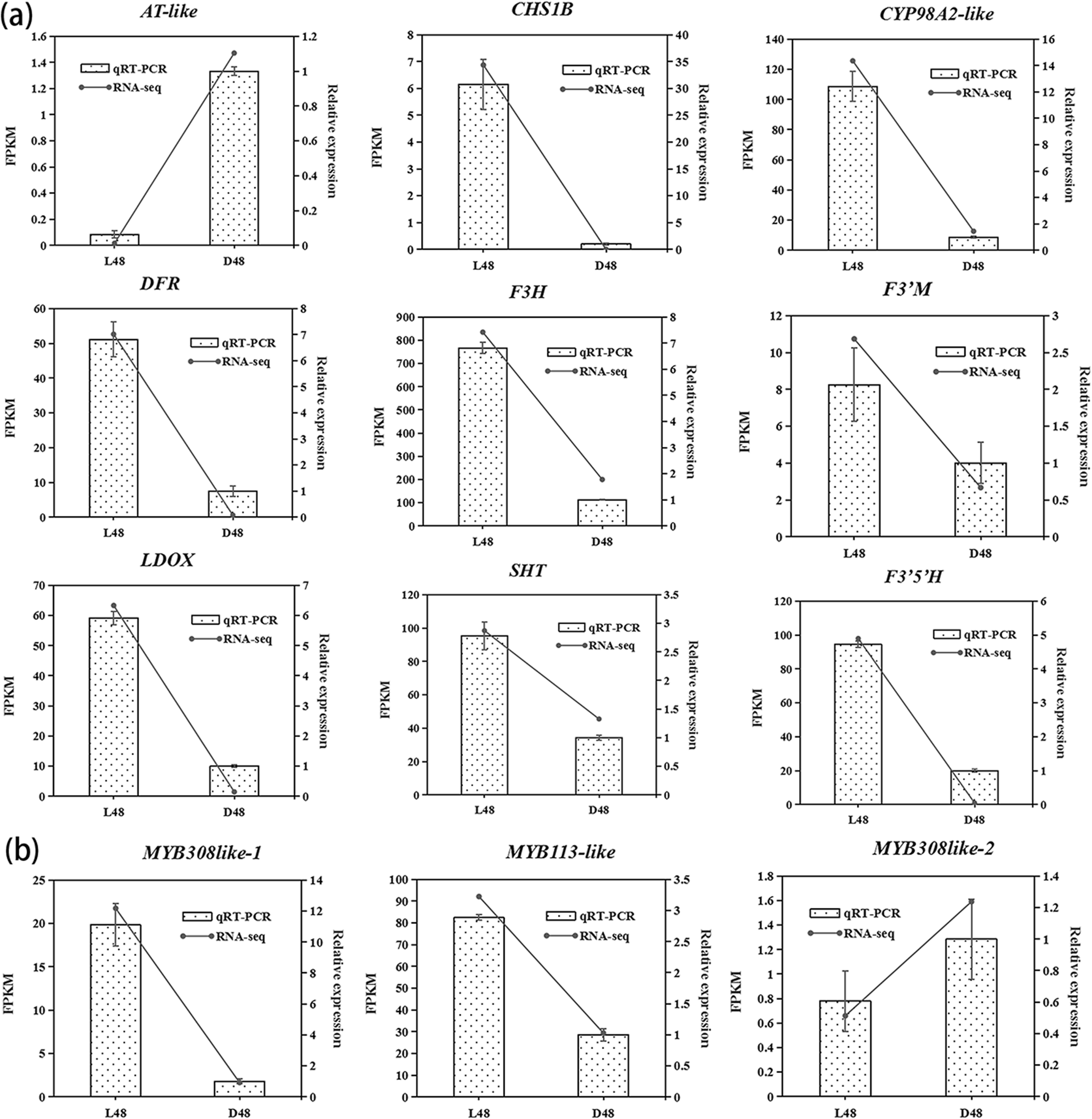

Supplement: Supplementary file 25 — Additional file 25: Figure S11. qRT-PCR analysis of genes related to anthocyanin metabolism of L48 and D48. a Relative expression analysis of synthetic genes. SHT (XM_016684802.1), AT-like (XM_016704776.1), CHS1B (XM_016710598.1), CYP98A2-like (XM_016688489.1), DFR (XM_016705224.1), F3’5’H (XM_016693437.1), F3H (XM_016705025.1), F3’M (XM_016707872.1), LDOX (XM_016712446.1). b Relative expression analysis of regulatory genes. MYB113-like (XM_016689220.1), MYB308like-1 (XM_016696983.1), MYB308like-2 (XM_016702244.1), The x-axis represents the different treatments, the y-axis represents the relative gene expression level and RNA-seq FPKM. Error bars indicate the standard error of the mean (n = 3). [file 12870_2021_3423_MOESM25_ESM.tif]
